# 

**Published:** 1879-10

**Authors:** 


### CONTENTS:

| | PAGE. |
|---|---|
| **Original Communications:** | |
| Perityphlitic Abscess, by Herman Mynter, M. D., | 85 |
| An Unrecognized Fracture of the Neck of the Femur, by Charles C. F. Gay, M. D., | 98 |
| **Clinical Reports:** | |
| Placenta Prævia, reported by John Hauenstein, M. D., | 103 |
| Remarkable Vitality, reported by Wm. H. Slacer, M. D., | 107 |
| A Case of Rupture of the Uterus, reported by P. W. Van Peyma, M. D., | 108 |
| **Selections:** | |
| The Brooklyn Treatment of Diphtheria, by Paul H. Kretzschmar, M. D., | 110 |
| New Paper Spinal Brace, by Morgan Vance, M. D. | 114 |
| The use of Hot Water in Hemorrhage following the Employment of Esmarch's Bandage, | 115 |
| Coto Bark, | 115 |
| Oxalate of Cerium in Pertussis, | 116 |
| Treatment of Vesical Atony by Ergotine Injections, | 116 |
| Syphilis by Vaccination with Human Virus, | 117 |
| Cholagogues, | 117 |
| Incontinence of Urine in Children, | 118 |
| **Society Reports:** | |
| Buffalo Medical Association, | 119 |
| **Editorial:** | |
| Medical Education, | 123 |
| Mortality Table, | 126 |
| Business Notice to Subscribers, | 127 |
| **Reviews,** | 128 |
| Books and Pamphlets received. | 132 |

